# Association between dietary intake of anthocyanidins and heart failure among American adults: NHANES (2007–2010 and 2017–2018)

**DOI:** 10.3389/fnut.2023.1107637

**Published:** 2023-04-05

**Authors:** Zaixiao Tao, Rui Zhang, Wenjie Zuo, Zhenjun Ji, Zhongguo Fan, Xi Chen, Rong Huang, Xinxin Li, Genshan Ma

**Affiliations:** ^1^Department of Cardiology, Zhongda Hospital, School of Medicine, Southeast University, Nanjing, China; ^2^School of Medicine, Southeast University, Nanjing, China

**Keywords:** anthocyanidins, heart failure, flavonoids, cardiovascular disease, NHANES

## Abstract

**Background:**

Despite anthocyanidins have anti-inflammatory and antioxidant properties, no studies have researched association between dietary intake of anthocyanidins and heart failure.

**Methods:**

We enrolled 15,869 participants from the National Health and Nutrition Examination Survey (NHANES) (2007–2010 and 2017–2018) in this cross-sectional study. We examined baseline data and prevalence of heart failure in different quartile groups of anthocyanin intake (Q1-4). Three models were established through logistic regression to evaluate the protective effect of Q4 (highest anthocyanidins intake) on heart failure. The protective effect of high anthocyanidins intake on heart failure was further evaluated in different subgroups.

**Results:**

Participants with the highest anthocyanidins intake (Q4) had the lowest prevalence of heart failure (Q1:2.54%, Q2:2.33%, Q3:2.43%, Q4:1.57%, *p* = 0.02). After adjusting for possible confounding factors, compared with the Q1 group, the highest anthocyanidins intake (Q4) was independently related to lower presence of heart failure (Q4: OR 0.469, 95%CI [0.289, 0.732], *p* = 0.003). And this association was still stable in subgroups of female, ≥45 years, smoker, non-Hispanic White or without diabetes, stroke and renal failure.

**Conclusion:**

Dietary intake of anthocyanidins had negative association with the presence of heart failure.

## Introduction

Heart failure (HF) is the terminal manifestation of cardiovascular disease (1). In recent years, the prevalence of HF has gradually increased, and its mortality and disability rate have also increased. The number of people with HF worldwide is predicted to be close to 64.3 million (2). Despite continuous progress in the treatment of HF, due to frequent hospital stays and ongoing treatment, patients with HF have severe everyday limits and bear a heavy financial burden (3). Moreover, 50% of people with HF with a decreased ejection fraction pass away within 5 years after being diagnosed (4). Thus, it is essential to inhibit the occurrence and development of HF.

With a focus on diet, people have realized that traditional western diet, such as red meat, high sugar food and fried food, are harmful to heart health (5), while omega-3 fatty acids (6), polyphenolic and flavonoids (7), as well as other micronutrients that are abundant in Indo-Mediterranean diets (8), may all play a protective role in maintaining the heart health. Anthocyanidins are one of the six major categories of flavonoids, and the anthocyanidins consumed in diet are mainly provided by fruits such as berries (9). Anthocyanidins have powerful anti-inflammatory and antioxidant properties, making them useful in the prevention of a variety of chronic diseases, such as eye and kidney complications and many cancer types (10–13). An increasing number of evidences show that anthocyanidins is related to circulatory disease, and have shown significant lipid-lowering effects in many studies (14, 15). Anthocyanidins also have positive effects on endothelial function and have antiatherogenic and anti-arterial stiffness properties (16). According to the meta-analysis, dietary anthocyanidins intake was linked to a lower risk of coronary heart disease and a lower mortality of cardiovascular diseases (17). Moreover, the link between anthocyanidins and cardiovascular diseases has been verified by numerous experimental research. Such as, anthocyanidins played a chemo-preventive role in atherosclerosis *via* activation of Nrf2-ARE pathway (18); through suppression of the ROS-JNK-Bcl-2 pathway, anthocyanidins reduces myocardial ischemia-induced damage (19).

However, the protective effect of anthocyanidins on HF has not been reported. Therefore, the purpose of this study was to assess the impact of dietary anthocyanidins on HF in the general American population.

## Materials and methods

### Study population

**Figure 1:**
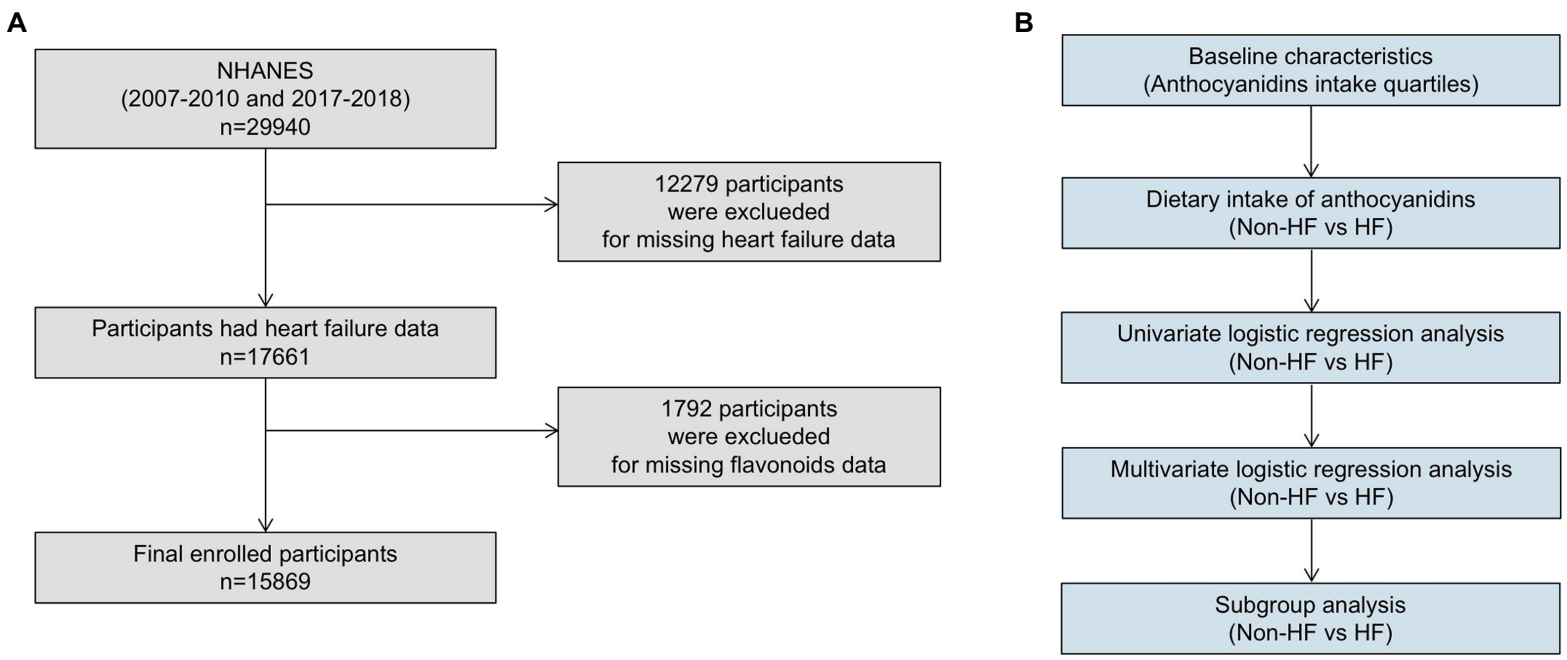
**(A)** Flow chart of participants selection; **(B)** flow chart of data analysis.

The National Health and Nutrition Examination Survey (NHANES) is a series of surveys designed on the basis of cross-sections to investigate the health status of all U.S. populations, which conducted by National Center for Health Statistics (NCHS). The survey included demographic information, dietary information, various physical examination indicators and health related data. All information and survey methods are available online.^1^ The NCHS Research Ethics Review Board authorized the research protocols and each participant signed a written statement of informed consent. Since only three NHANES circles (2007–2008, 2009–2010 and 2017–2018) investigated the dietary intake of flavonoids, this study included an investigation of those three NHANES circles. Exclusion criteria included: age <18 years; missing HF status; missing dietary information about flavonoids (Figure 1).

### Assessment of dietary anthocyanidins intakes

This study mainly collects the intake of flavonoids in foods and beverages, which are usually onions, potatoes, celery, etc. According to the food code from Nutrient Database for Dietary Studies (FNDDS), the food types were refined. Different codes represent different flavonoid contents. Versions of the FNDDS that are suitable for each survey cycle were utilized: version 4.1 was used for 2007–2008, while version 5.0 was used for 2009–2010 and 2017–2018 (20). Six of the flavonoid classes (anthocyanidins, flavan-3-ols, flavanones, flavones, flavonols and isoflavones) as well as the total daily intake of all flavonoids (the sum of the 29 individual flavonoids) were calculated from all foods and beverages.

### Assessment of HF

Like previous NHANES-based articles that have been published (21), participants were asked in the health questionnaires “whether a doctor or other health professional has ever told you that you had heart failure” and those who responded “yes” were considered to have HF.

### Covariates

NHANES collected demographic data on all participants. Race was divided into four categories, non-Hispanic White, non-Hispanic Black, Mexican American, and others. Smoking was divided into two categories: yes (now, former) and no (never). Diabetes, hypertension and hyperlipidemia were all diagnosed by doctors. The systolic and diastolic blood pressure, body mass index (BMI), and waist measured by experts using conventional physical examination techniques. In a typical laboratory, the level of triglycerides (TG), total cholesterol (TC), high-density cholesterol (HDL), low-density cholesterol (LDL), fasting plasma glucose (FPG), hemoglobin A1c (HbA1c), and creatinine were all measured. We calculated the estimated glomerular filtration rate (eGFR) through the creatinine equation. Information about the specific techniques and quality of the determination of all covariate control methods were accessible from Website of NHANES.

### Statistical analysis

For all statistical studies, R Programming Language (version 4.2.1) were used. Statistical significance was determined to two-tailed, *p* < 0.05. Analysis method was referred to previously NHANES-based articles (22). Participants were divided into four groups (Q1-4) according to the quartile of anthocyanidins intake. We adjusted the weights in our analysis to prevent oversampling and lower the non-response rate. Weighted means (95% confidence intervals [CIs]) and weighted percentages (95% CIs) were used to describe continuous variables and categorical variables, respectively. To evaluate differences between groups, the categorical variables used a weighted chi-square test and the continuous variables used a weighted linear regression model. Univariable and multivariable logistic regression models were used to analyze the connections between HF and anthocyanidins consumption in all participants and different subgroups (Figure 1).

## Results

### Baseline characteristics of study population

**Figure 2:**
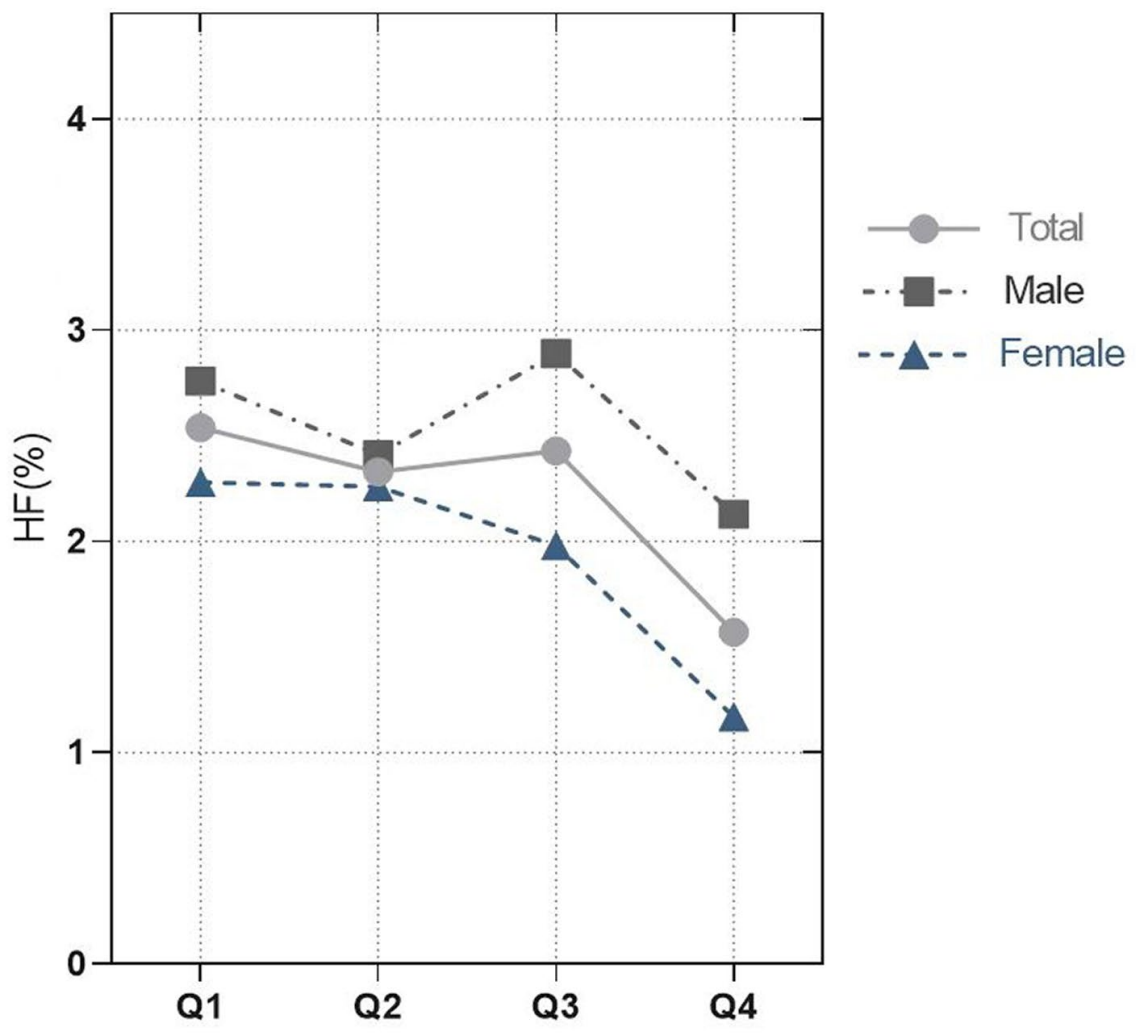
Proportion of heart failure in the quartile of anthocyanidins.

This study enrolled 15,869 participants which including 513 (3.23%) HF patients (Figure 1). The average age of all participants was 47.40 (46.81, 47.99) years old, including 48.65% men and 51.35% women. The baseline characteristics are shown based on the dietary anthocyanidins intake quartiles (Q1: 0 mg; Q2: [0, 0.73] mg; Q3: [0.73, 6.29] mg and Q4: >6.29 mg) (Table 1). Compared to the other quartiles, individual who divided in Q4 group were likely to be older, female, non-Hispanic White, receive medications of statin. Regarding the traditional risk factors for cardiovascular disease, the Q4 group had increased levels of HDL cholesterol but lower levels of BMI, waist, triglycerides, and diastolic pressure. Most importantly, the prevalence of HF was lower in the Q4 group (Q1:2.54%, Q2:2.33%, Q3:2.43%, Q4:1.57%, *p* = 0.02) (Figure 2), but there is no significant difference in the prevalence of coronary heart disease, stroke and diabetes.

**Table 1:** Baseline characteristics of participants.

Variable	Total	Q1	Q2	Q3	Q4	*p* value
Count	15,869 (100.00)	5,598 (35.28)	2,343 (14.76)	3,961 (24.96)	3,967 (25)	
Total Anthocyanidins, mg	13.72 (12.41,15.02)	0.00(0.00, 0.00)	0.24(0.22, 0.25)	2.82(2.75, 2.88)	47.59 (44.47,50.71)	<0.0001
Age	47.40 (46.81,47.99)	44.39 (43.73,45.04)	47.10 (45.98,48.22)	48.87 (48.06,49.68)	50.18 (49.24,51.11)	<0.0001
Gender						<0.0001
Male	7,720 (48.65)	2,960 (53.29)	1,086 (46.66)	1916 (49.01)	1758 (42.10)	
Female	8,149 (51.35)	2,638 (46.71)	1,257 (53.34)	2045 (50.99)	2,209 (57.90)	
Race						<0.0001
White	7,007 (44.16)	2,504 (67.08)	1,003 (66.12)	1,550 (62.93)	1950 (71.56)	
Black	3,287 (20.71)	1,390 (13.90)	465 (10.39)	783 (11.60)	649 (8.27)	
Mexican	2,592 (16.33)	751 (7.17)	435 (10.35)	838 (10.92)	568 (6.99)	
Other	2,983 (18.8)	953 (11.85)	440 (13.14)	790 (14.55)	800 (13.18)	
Smoke						<0.0001
No	8,652 (54.52)	2,678 (48.00)	1,319 (57.40)	2,256 (57.86)	2,399 (61.84)	
Yes	7,216 (45.48)	2,920 (52.00)	1,023 (42.60)	1705 (42.14)	1,568 (38.16)	
Body mass index, kg/m2	29.09 (28.86,29.32)	29.82 (29.51,30.13)	29.12 (28.65,29.59)	29.00 (28.67,29.33)	28.22 (27.90,28.53)	<0.0001
Waist, cm	99.13 (98.48,99.77)	100.93 (100.04,101.82)	99.04 (97.97,100.11)	98.94 (97.98, 99.91)	97.01 (96.27, 97.75)	<0.0001
Systolic pressure, mmHg	121.67 (121.14,122.19)	122.13 (121.39,122.87)	121.23 (120.29,122.17)	122.10 (121.11,123.10)	120.95 (120.12,121.78)	0.09
Diastolic pressure, mmHg	71.09 (70.46,71.72)	71.84 (70.98,72.70)	71.35 (70.50,72.21)	70.76 (70.05,71.46)	70.28 (69.60,70.95)	0.001
Triglycerides, mg/dl	152.81 (149.11,156.51)	154.89 (148.77,161.01)	155.22 (149.07,161.37)	161.31 (155.62,167.00)	141.77 (137.59,145.96)	<0.0001
Total cholesterol, mg/dl	194.43 (192.89,195.98)	192.81 (190.39,195.23)	195.28 (192.64,197.92)	195.22 (193.52,196.92)	195.38 (193.01,197.75)	0.19
HDL cholesterol, mg/dl	52.97 (52.42,53.52)	50.92 (50.20,51.64)	52.82 (51.85,53.79)	52.45 (51.69,53.21)	56.10 (55.26,56.94)	<0.0001
LDL cholesterol, mg/dl	114.31 (112.96,115.67)	113.88 (111.40,116.35)	115.99 (112.66,119.32)	114.83 (112.79,116.87)	113.58 (111.18,115.98)	0.72
FPG, mmol/L	5.95 (5.89,6.01)	5.94 (5.86,6.02)	5.96 (5.85,6.08)	5.97 (5.87,6.07)	5.94 (5.82,6.07)	0.94
Hemoglobin A1c, %	5.63 (5.61,5.66)	5.62 (5.60,5.65)	5.63 (5.59,5.67)	5.68 (5.63,5.73)	5.60 (5.57,5.64)	0.02
eGFR, mL/min/1.73 m^2^	94.64 (93.71,95.56)	96.92 (95.87,97.97)	94.74 (93.24,96.25)	94.01 (92.90,95.12)	92.20 (90.91,93.48)	<0.0001
Creatinine, mg/dl	0.88 (0.87,0.89)	0.89 (0.88,0.91)	0.87 (0.86,0.89)	0.88 (0.87,0.89)	0.87 (0.86,0.88)	0.004
**Diseases**						
Heart failure	513 (3.23)	201 (2.54)	78 (2.33)	137 (2.43)	97 (1.57)	0.02
DM						0.23
DM	3,080 (19.62)	1,058 (14.10)	454 (13.93)	852 (15.96)	716 (13.49)	
IFG	814 (5.19)	311 (5.56)	114 (5.60)	184 (4.51)	205 (5.39)	
IGT	474 (3.02)	140 (2.19)	77 (2.76)	130 (2.94)	127 (3.05)	
No	11,328 (72.17)	4,042 (78.14)	1,668 (77.71)	2,756 (76.59)	2,862 (78.07)	
Hypertension	6,933 (43.69)	2,455 (38.38)	1,042 (37.38)	1765 (38.67)	1,671 (35.93)	0.33
Hyperlipidemia	11,227 (70.76)	3,917 (68.76)	1,659 (70.66)	2,858 (70.49)	2,793 (67.79)	0.27
Coronary heart disease	666 (4.21)	226 (3.19)	90 (3.17)	155 (3.36)	195 (4.20)	0.06
Stroke	702 (4.43)	252 (3.08)	132 (3.92)	174 (3.45)	144 (2.68)	0.16
**Medications**						
ACE inhibitors	386 (2.43)	125 (1.78)	71 (2.66)	102 (2.18)	88 (1.95)	0.31
Beta blocker	2,153 (13.58)	713 (10.29)	329 (12.17)	554 (12.39)	557 (11.40)	0.13
Diuretics	2,319 (14.62)	760 (10.40)	365 (12.90)	617 (12.59)	577 (11.51)	0.1
Statin	3,125 (19.71)	957 (13.91)	501 (17.30)	845 (19.02)	822 (18.00)	<0.0001

eGFR, glomerular filtration rate; HDL cholesterol, high-density cholesterol; LDL cholesterol, low-density cholesterol; FBG, fasting plasma glucose; DM, diabetes mellitus; IFG, impaired fasting glucose; IGT, impaired glucose tolerance.

### Association between dietary anthocyanidins intake and HF

We compared the dietary intake of flavonoids between non-HF and HF. Surprisingly, although there was no difference in the intake of total flavonoids and other five flavonoids, the intake of anthocyanidins in non-HF was higher than that in HF (13.84 [12.52, 15.17] mg vs. 8.04 [5.81, 10.28] mg; *p* < 0.0001). Moreover, the five anthocyanidins subclasses (cyanidin, delphinidin, petunidin, malvidin, peonidin) also showed the same trends (Table 2). In Supplementary Table S1, the findings of univariate logistic regression analyses for HF were shown. The dietary anthocyanidins intake were negatively correlated with HF. Conversely, risk factors for cardiovascular diseases, such as age, smoke, BMI, waist, triglycerides, fasting plasma glucose were positively with HF. Compared to the Q1 group, participants with highest dietary anthocyanidins (Q4) intake showed a lower presence of HF (OR 0.61, 95% CI [0.46–0.81]; *p* < 0.001) in the unadjusted model. Table 3 displayed the findings of multivariate logistic regression analysis for the relationship between dietary anthocyanidins intake and HF. Highest dietary anthocyanidins intake (Q4) was independently associated with lower presence of HF with adjustment for age, sex, race, smoke, BMI, waist, systolic pressure, diastolic pressure, diabetes, hypertension, hyperlipidemia, coronary heart disease, stroke, ACE inhibitor, Beta blocker, diuretics, statin, eGFR, creatinine, HbAlc, FPG, HDL, LDL, TC, TG (OR 0.467, 95% CI [0.302, 0.751]; *p* = 0.003). Additionally, we transformed intake of dietary anthocyanidins into a categorical variable (Q1-4), both the unadjusted (*p* for trend <0.001) and adjusted (*p* for trend = 0.005) models showed significant *p* for trends.

**Table 2:** Flavonoid in patients with or without heart failure.

Variable	Total	Non-HF	HF	*p* value
Total anthocyanidins, mg	13.72 (12.41, 15.02)	13.84 (12.52, 15.17)	8.04 (5.81, 10.28)	<0.0001
Total isoflavones, mg	2.01 (1.70, 2.32)	2.02 (1.71, 2.34)	1.55 (0.68, 2.42)	0.29
Total flavan-3-ols, mg	186.91 (171.93, 201.89)	186.93 (171.76, 202.10)	186.01 (127.14, 244.88)	0.98
Total flavanones, mg	12.27 (11.51, 13.02)	12.28 (11.52, 13.03)	11.90 (9.10, 14.70)	0.79
Total flavonols, mg	19.52 (18.79, 20.25)	19.58 (18.84, 20.32)	16.89 (14.13, 19.66)	0.07
Total flavones, mg	0.92 (0.86, 0.97)	0.92 (0.87, 0.97)	0.79 (0.46, 1.13)	0.46
Total sum of all flavonoids, mg	235.35 (219.62, 251.07)	235.58 (219.63, 251.52)	225.19 (163.24, 287.13)	0.75
**Anthocyanidins**				
Cyanidin, mg	2.55 (2.20, 2.89)	2.57 (2.22, 2.92)	1.61 (1.13, 2.09)	<0.001
Delphinidin, mg	1.76 (1.30, 2.21)	1.78 (1.32, 2.25)	0.66 (0.35, 0.97)	<0.0001
Petunidin, mg	1.13 (0.96, 1.30)	1.14 (0.97, 1.32)	0.55 (0.27, 0.82)	<0.0001
Malvidin, mg	4.75 (4.24, 5.26)	4.80 (4.28, 5.31)	2.72 (1.66, 3.78)	<0.001
Peonidin, mg	1.94 (1.65, 2.22)	1.96 (1.67, 2.24)	1.04 (0.58, 1.49)	0.001
Pelargonidin, mg	1.60 (1.37, 1.83)	1.60 (1.37, 1.83)	1.47 (0.72, 2.22)	0.75

**Table 3:** Associations between total anthocyanidins and heart failure.

	Unadjusted model	Model 1	Model 2	Model 3
Character	95%CI, *p*	OR 95%CI, *p*	OR 95%CI, *p*	OR 95%CI, *p*
Q1	ref	ref	ref	ref
Q2	0.919 (0.644, 1.310)	0.780 (0.540, 1.128)	0.800 (0.551, 1.160)	0.583 (0.282, 1.205)
	0.633	0.182	0.232	0.112
Q3	0.957 (0.699, 1.310)	0.708 (0.513, 0.978)	0.728 (0.527, 1.004)	0.779 (0.465, 1.307)
	0.777	0.037	0.053	0.321
Q4	0.614 (0.465, 0.811)	0.426 (0.324, 0.562)	0.446 (0.336, 0.591)	0.467 (0.302, 0.751)
	<0.001	<0.001	<0.001	0.003
*p* for trend	<0.001	<0.001	<0.001	0.005

Unadjusted model: univariate logistic regression analyses. Model 1 was adjusted for age, sex. Model 2 was adjusted for variables included in Model 1 and race. Model 3 was adjusted for variables included in Model 1 and smoke, body mass index, waist, systolic pressure, diastolic pressure, diabetes, hypertension, hyperlipidemia, coronary heart disease, stroke, ACE inhibitor, Beta blocker, diuretics, statin, eGFR, creatinine, hemoglobin A1c, fast glucose, HDL cholesterol, LDL cholesterol, total cholesterol, triglycerides. OR, odd ratio; CI, confidence interval.

### Subgroup analyses

**Figure 3:**
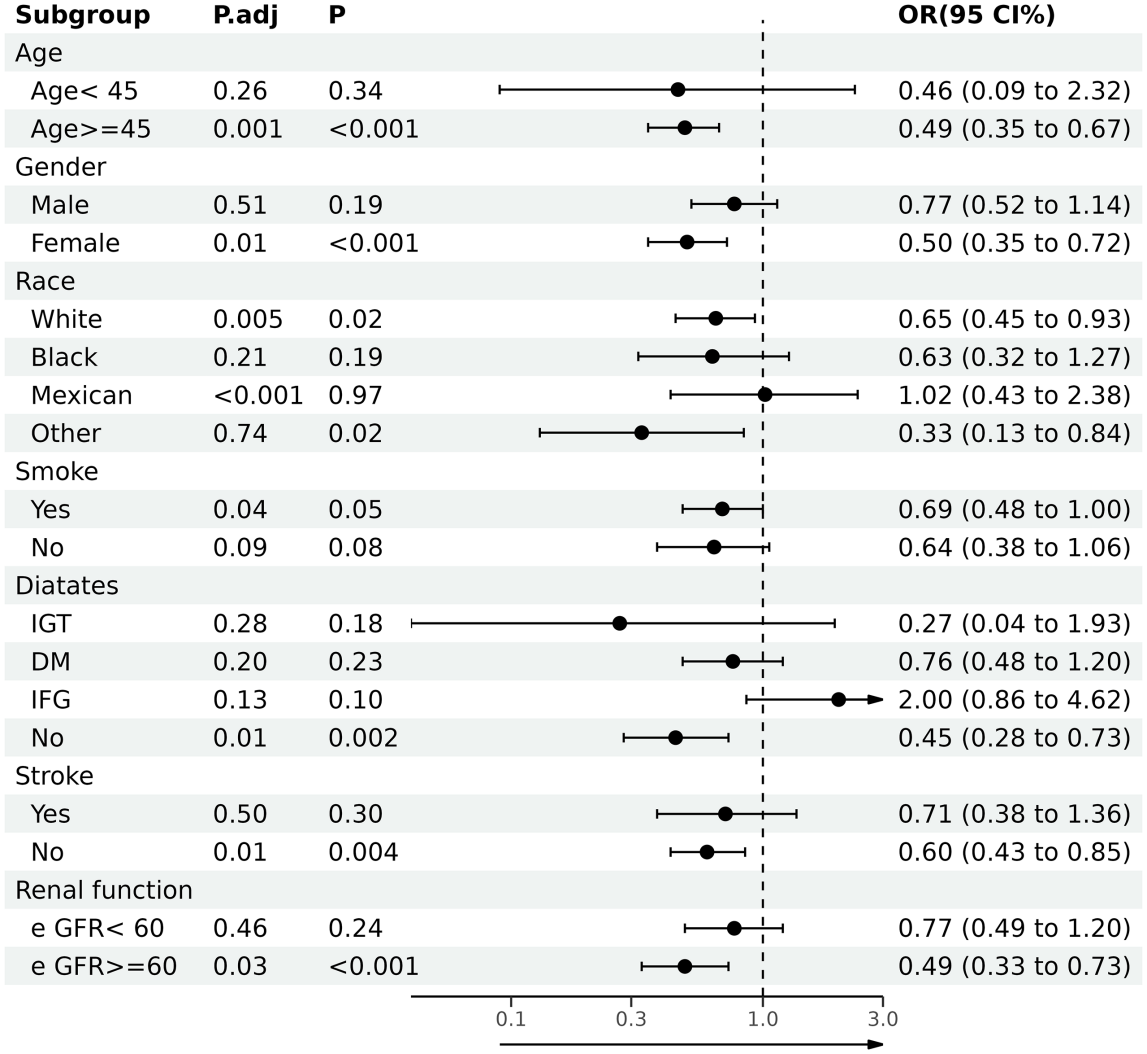
Association between total anthocyanidins (Q4) and heart failure in various stratifications. OR, odd ratio; CI, confidence interval; DM, diabetes mellitus; IFG, impaired fasting glucose; IGT, impaired glucose tolerance; eGFR, glomerular filtration rate.

Through subgroup analysis, we further investigate the correlation between dietary anthocyanidins intake and HF in different populations (Figure 3). The whole population was stratified by age, sex, race and different disease status. In the subgroups of ≥45 years (OR 0.49, 95% CI [0.35, 0.67]), female (OR 0.50, 95% CI [0.35, 0.72]), smoker (OR 0.69, 95% CI [0.48, 1.00]), non-Hispanic White (OR 0.65, 95% CI [0.45, 0.93]) or without diabetes (OR 0.45, 95% CI [0.28, 0.73]), stroke (OR 0.60, 95% CI [0.43, 0.85]), renal failure (OR 0.49, 95% CI [0.33.0.73]), this association was still stable. Furthermore, we conducted independent multivariate logistic regression analysis for each subgroup. The variables enrolled in model3 were all retained in this analysis except for the variables that were used for stratification. These trends were consistent with before.

## Discussion

As far as we know, this study was the first time to demonstrate the association between dietary anthocyanidins intake and HF, dietary anthocyanidins consumption (categorical) and HF were found to be negatively correlated in the NHANES 2007–2010 and 2017–2018. When dietary anthocyanidins intake was in the Q4 group, the incidence of HF was lowered by 50% after adjusting probable confounding factors. This negative correlation is still stable in subgroups of female, ≥45 years, former smoker, non-Hispanic White or without diabetes, stroke, renal failure. Moreover, compared with anthocyanidins intake between HF and non-HF for each quartile, it was found that only anthocyanins intake of Q4 was different between HF and non-HF, the intake of anthocyanins in non-HF was higher than that in HF (Supplementary Figure S1; Supplementary Table S2).

At present, cardiovascular disease has caused great social burden, so many scholars are emphasizing the importance of dietary habits in the prevention and treatment of cardiovascular diseases (23). With the increasing demand for flavonoids (24), anthocyanidins have been discovered many benefits for cardiovascular system, which is a subgroup of flavonoids (25). As some studies have shown, a high dietary intake of anthocyanidins was linked to decreased total cardiovascular disease incidence and mortality. For example, Adriouch S et al. found that participants in the highest tertiles of anthocyanidins had a 34% lower risk of major cardiovascular events than those in the lowest tertiles following multivariable adjustment (26). Besides, Lin Xu et al. confirmed that dietary intake of anthocyanidins significantly decreased the risk of death from all cardiovascular diseases in meta-analysis including 2,36,648 subjects and 9,765 cases (RR: 0.91, 95% CI [0.87, 0.96]; *p* < 0.001), it was also found that dietary anthocyanidins may have a more significant protective effect on total CVD mortality in women (27). Additionally, dietary anthocyanidins intake is also beneficial in vascular diseases. Such as, Margarethe E Goetz et al. reported that anthocyanidins intake was negatively correlated with incident of coronary heart disease after matching age, sex, race and residence (28). Moreover, anthocyanidins consumption in the diet is crucial for preventing subclinical injury of cardiovascular disease, such as hyperlipidemia, obesity, vascular endothelial function, arterial stiffness, decreased cardiac systolic function (29–32) Although some studies believed that purified anthocyanidins had more cardioprotective effects than dietary anthocyanidins (27), this conclusion is still controversial due to incompleteness of dietary data and differences in interventions (33–36). The evidence for the benefits of dietary anthocyanidins on cardiovascular system is very strong, but our research revealed for the first time a connection between dietary anthocyanidins intake and the presence of HF in the general population.

Although our clinical studies revealed an association between dietary intake of anthocyanidins and HF, the underlying mechanism had not been clarified. Thus, we summarized the following four possible mechanisms through literature summary. First, oxidative stress disorder plays a crucial role in the in the occurrence and development of HF. When the production of reactive oxygen species (ROS) exceeds the internal defense capacity of cells, excess ROS will attack cells, leading to protein and lipid peroxidation, DNA damage, and ultimately cell death (37). Anthocyanidins, a naturally occurring plant pigment, not only serves as a colorant but also has strong antioxidant properties, ROS such superoxide anion, singlet oxygen, and peroxide free radical can be neutralized by anthocyanidins (38). Second, in the pathophysiological process of chronic HF, a long-lasting inflammatory response causes adverse ventricular remodeling (39). The anti-inflammatory capabilities of anthocyanidins have also been proven in numerous research (40–44), anthocyanidins can inhibit NF-κB activity to reduce inflammation level. Third, some clinical studies have found that anthocyanidins can improve cardiovascular metabolic disorder and obesity, which are high risk factors for HF (29, 30). Fourth, rich-anthocyanidins foods also contain dietary fiber, vitamins and various polyphenols, which also have certain protective effects on heart health (45–48). To investigate established and speculative mechanisms, further basic and clinical research is required.

Nevertheless, there were some limitations in this study. First, as a cross-sectional study, this study was unable to confirm the causal relationship between dietary intake of anthocyanidins and HF. Second, this study only evaluated the effect of anthocyanidins in food, but whether the purified anthocyanidins had the same effect still needs further randomized controlled experiments. Third, the NHANES database does not provide brain natriuretic peptide and echocardiography data, so this study cannot further evaluate the relationship between dietary intake of anthocyanidins and the severity of HF. Moreover, uncontrollable confounding variables may also need further analysis, such as physical activity and nutritional supplements. Finally, the subjects of this study were adult Americans, excluding adolescents and children, which would affect the promotion of the research results.

## Conclusion

In conclusion, dietary intake of anthocyanidins was associated with HF negatively, people can decrease the presence of HF by increasing anthocyanidins in their daily diets. To determine their clear relationship, more cellular, animal, and human investigations are necessary.

## Data availability statement

The datasets presented in this study can be found in online repositories. The names of the repository/repositories and accession number(s) can be found in the article/Supplementary material.

## Ethics statement

The studies involving human participants were reviewed and approved by National Health and Nutrition Examination Survey (NHANES). The patients/participants provided their written informed consent to participate in this study. Written informed consent was obtained from the individual(s) for the publication of any potentially identifiable images or data included in this article.

## Author contributions

ZT conceived and designed the study. RZ, WZ, and ZJ were responsible for the management and retrieval of data, contributed to initial data analysis, and interpretation. ZT drafted the initial manuscript. XC, RH, and XL revised the manuscript and were the guarantors of this work and had full access to all the data in the study. GM take responsibility for its integrity and the accuracy of the data analysis. All authors contributed to the article and approved the submitted version.

## Conflict of interest

The authors declare that the research was conducted in the absence of any commercial or financial relationships that could be construed as a potential conflict of interest.

## Publisher’s note

All claims expressed in this article are solely those of the authors and do not necessarily represent those of their affiliated organizations, or those of the publisher, the editors and the reviewers. Any product that may be evaluated in this article, or claim that may be made by its manufacturer, is not guaranteed or endorsed by the publisher.

## Footnotes

^1^ https://www.cdc.gov/nchs/nhanes/index.htm

## References

[1] Metra M Teerlink JR. Heart failure. Lancet. (2017) 390:1981–95. doi: 10.1016/s0140-6736(17)31071-1, PMID: 28460827

[2] GBD 2017 Disease and Injury Incidence and Prevalence Collaborators. Global, regional, and national incidence, prevalence, and years lived with disability for 354 diseases and injuries for 195 countries and territories, 1990-2017: a systematic analysis for the global burden of disease study 2017. Lancet. (2018) 392:1789–858. doi: 10.1016/s0140-6736(18)32279-7, PMID: 30496104 PMC6227754

[3] Dunlay SM Roger VL. Understanding the epidemic of heart failure: past, present, and future. Curr Heart Fail Rep. (2014) 11:404–15. eng. The authors have no disclosures or potential conflicts of interest. doi: 10.1007/s11897-014-0220-x, PMID: 25182014 PMC4224604

[4] Go AS Mozaffarian D Roger VL Benjamin EJ Berry JD Blaha MJ. Executive summary: heart disease and stroke statistics-2014 update: a report from the American Heart Association. Circulation. (2014) 129:399–410. doi: 10.1161/01.cir.0000442015.53336.12, PMID: 24446411

[5] Singh RB Fedacko J Pella D Fatima G Elkilany G Moshiri M. High exogenous antioxidant, restorative treatment (heart) for prevention of the six stages of heart failure: the heart diet. Antioxidants (Basel). (2022) 11:1464. doi: 10.3390/antiox11081464, PMID: 36009183 PMC9404840

[6] Bassuk SS Manson JE. Marine omega-3 fatty acid supplementation and prevention of cardiovascular disease: update on the randomized trial evidence. Cardiovasc Res. (2022): 00:1–13. doi: 10.1093/cvr/cvac172, PMID: 36378553 PMC10262192

[7] Parmenter BH Dalgaard F Murray K Marquis-Gravel G Cassidy A Bondonno CP. Intake of dietary flavonoids and incidence of ischemic heart disease in the Danish diet, cancer, and health cohort. Eur J Clin Nutr. (2023) 77:270–7. doi: 10.1038/s41430-022-01226-y, PMID: 36284213 PMC9908533

[8] Singh RB Fedacko J Fatima G Magomedova A Watanabe S Elkilany G. Why and how the indo-Mediterranean diet may be superior to other diets: the role of antioxidants in the diet. Nutrients. (2022) 14:898. doi: 10.3390/nu14040898, PMID: 35215548 PMC8879532

[9] Sebastian RS Wilkinson Enns C Goldman JD Martin CL Steinfeldt LC Murayi T. A new database facilitates characterization of flavonoid intake, sources, and positive associations with diet quality among US adults. J Nutr. (2015) 145:1239–48. doi: 10.3945/jn.115.213025, PMID: 25948787 PMC4442120

[10] Roy P Tomassoni D Traini E Martinelli I Micioni Di Bonaventura MV Cifani C. Natural antioxidant application on fat accumulation: preclinical evidence. Antioxidants (Basel). (2021) 10:858. doi: 10.3390/antiox10060858, PMID: 34071903 PMC8227384

[11] Speciani MC Cintolo M Marino M Oren M Fiori F Gargari G. Flavonoid intake in relation to colorectal cancer risk and blood bacterial DNA. Nutrients. (2022) 14:4516. doi: 10.3390/nu14214516, PMID: 36364779 PMC9653960

[12] Ng D Altamirano-Vallejo JC Gonzalez-De la Rosa A Navarro-Partida J Valdez-Garcia JE Acosta-Gonzalez R. An oral polyphenol formulation to modulate the ocular surface inflammatory process and to improve the symptomatology associated with dry eye disease. Nutrients. (2022) 14:3236. doi: 10.3390/nu14153236, PMID: 35956412 PMC9370512

[13] Li YX Lu YP Tang D Hu B Zhang ZY Wu HW. Anthocyanin improves kidney function in diabetic kidney disease by regulating amino acid metabolism. J Transl Med. (2022) 20:510. doi: 10.1186/s12967-022-03717-9, PMID: 36335368 PMC9636632

[14] Liang Y Chen J Zuo Y Ma KY Jiang Y Huang Y. Blueberry anthocyanins at doses of 0.5 and 1% lowered plasma cholesterol by increasing fecal excretion of acidic and neutral sterols in hamsters fed a cholesterol-enriched diet. Eur J Nutr. (2013) 52:869–75. doi: 10.1007/s00394-012-0393-6 22684634

[15] Yang L Ling W Du Z Chen Y Li D Deng S. Effects of Anthocyanins on Cardiometabolic health: a systematic review and meta-analysis of randomized controlled trials. Adv Nutr. (2017) 8:684–93. doi: 10.3945/an.116.014852, PMID: 28916569 PMC5593100

[16] Mozos I Flangea C Vlad DC Gug C Mozos C Stoian D. Effects of Anthocyanins on vascular health. Biomol Ther. (2021) 11:811. doi: 10.3390/biom11060811, PMID: 34070757 PMC8227852

[17] Kimble R Keane KM Lodge JK Howatson G. Dietary intake of anthocyanins and risk of cardiovascular disease: a systematic review and meta-analysis of prospective cohort studies. Crit Rev Food Sci Nutr. (2019) 59:3032–43. doi: 10.1080/10408398.2018.1509835, PMID: 30277799

[18] Aboonabi A Singh I. Chemopreventive role of anthocyanins in atherosclerosis via activation of Nrf2-ARE as an indicator and modulator of redox. Biomed Pharmacother. (2015) 72:30–6. doi: 10.1016/j.biopha.2015.03.008, PMID: 26054672

[19] Syeda MZ Fasae MB Yue E Ishimwe AP Jiang Y Du Z. Anthocyanidin attenuates myocardial ischemia induced injury via inhibition of ROS-JNK-Bcl-2 pathway: new mechanism of anthocyanidin action. Phytother Res. (2019) 33:3129–39. doi: 10.1002/ptr.6485, PMID: 31774233

[20] Sebastian RS Wilkinson Enns C Goldman JD Moshfegh AJ. Dietary flavonoid intake is inversely associated with cardiovascular disease risk as assessed by body mass index and waist circumference among adults in the United States. Nutrients. (2017) 9:827. doi: 10.3390/nu9080827 28767062 PMC5579620

[21] Zhang X Sun Y Li Y Wang C Wang Y Dong M. Association between visceral adiposity index and heart failure: a cross-sectional study. Clin Cardiol. (2023) 6:310–9. doi: 10.1002/clc.23976, PMID: 36651220 PMC10018101

[22] Cai J Zhang L Chen C Ge J Li M Zhang Y. Association between serum Klotho concentration and heart failure in adults, a cross-sectional study from NHANES 2007-2016. Int J Cardiol. (2023) 370:236–43. doi: 10.1016/j.ijcard.2022.11.010, PMID: 36351541

[23] Adhikary D Barman S Ranjan R Stone H. A systematic review of major cardiovascular risk factors: a growing Global Health concern. Cureus. (2022) 14:e30119. doi: 10.7759/cureus.30119, PMID: 36381818 PMC9644238

[24] Roe AL Venkataraman A. The safety and efficacy of botanicals with Nootropic effects. Curr Neuropharmacol. (2021) 19:1442–67. doi: 10.2174/1570159x19666210726150432, PMID: 34315377 PMC8762178

[25] Dong Y Wu X Han L Bian J He C El-Omar E. The potential roles of dietary Anthocyanins in inhibiting vascular endothelial cell senescence and preventing cardiovascular diseases. Nutrients. (2022) 14:2836. doi: 10.3390/nu14142836, PMID: 35889793 PMC9316990

[26] Adriouch S Lampuré A Nechba A Baudry J Assmann K Kesse-Guyot E. Prospective association between Total and specific dietary polyphenol intakes and cardiovascular disease risk in the Nutrinet-Santé French cohort. Nutrients. (2018) 10:1587. doi: 10.3390/nu10111587, PMID: 30380657 PMC6266343

[27] Xu L Tian Z Chen H Zhao Y Yang Y. Anthocyanins anthocyanin-rich berries, and cardiovascular risks: systematic review and meta-analysis of 44 randomized controlled trials and 15 prospective cohort studies. Front Nutr. (2021) 8:747884. doi: 10.3389/fnut.2021.747884, PMID: 34977111 PMC8714924

[28] Goetz ME Judd SE Safford MM Hartman TJ McClellan WM Vaccarino V. Dietary flavonoid intake and incident coronary heart disease: the REasons for geographic and racial differences in stroke (REGARDS) study. Am J Clin Nutr. (2016) 104:1236–44. doi: 10.3945/ajcn.115.129452, PMID: 27655439 PMC5081714

[29] Daneshzad E Shab-Bidar S Mohammadpour Z Djafarian K. Effect of anthocyanin supplementation on cardio-metabolic biomarkers: a systematic review and meta-analysis of randomized controlled trials. Clin Nutr. (2019) 38:1153–65. doi: 10.1016/j.clnu.2018.06.979, PMID: 30007479

[30] Lee YM Yoon Y Yoon H Park HM Song S Yeum KJ. Dietary anthocyanins against obesity and inflammation. Nutrients. (2017) 9:1089. doi: 10.3390/nu9101089, PMID: 28974032 PMC5691706

[31] Arisi TOP Gorski F Eibel B Barbosa E Boll L Waclawovsky G. Dietary intake of anthocyanins improves arterial stiffness, but not endothelial function, in volunteers with excess weight: a randomized clinical trial. Phytother Res. (2022) 37:798–808. doi: 10.1002/ptr.7659, PMID: 36206152

[32] Cook MD Dunne A Bosworth M Willems MET. Effect of intake duration of anthocyanin-rich New Zealand blackcurrant extract on cardiovascular responses and femoral artery diameter during sustained submaximal isometric contraction. J Diet Suppl. (2023) 20:15–27. doi: 10.1080/19390211.2021.1948943, PMID: 35404735

[33] McAnulty SR McAnulty LS Morrow JD Khardouni D Shooter L Monk J. Effect of daily fruit ingestion on angiotensin converting enzyme activity, blood pressure, and oxidative stress in chronic smokers. Free Radic Res. (2005) 39:1241–8. doi: 10.1080/10715760500306836, PMID: 16298751

[34] Duthie SJ Jenkinson AM Crozier A Mullen W Pirie L Kyle J. The effects of cranberry juice consumption on antioxidant status and biomarkers relating to heart disease and cancer in healthy human volunteers. Eur J Nutr. (2006) 45:113–22. doi: 10.1007/s00394-005-0572-9, PMID: 16032375

[35] Nyberg S Gerring E Gjellan S Vergara M Lindström T Nystrom FH. Effects of exercise with or without blueberries in the diet on cardio-metabolic risk factors: an exploratory pilot study in healthy subjects. Ups J Med Sci. (2013) 118:247–55. doi: 10.3109/03009734.2013.825348, PMID: 23977864 PMC4190883

[36] McAnulty LS Collier SR Landram MJ Whittaker DS Isaacs SE Klemka JM. Six weeks daily ingestion of whole blueberry powder increases natural killer cell counts and reduces arterial stiffness in sedentary males and females. Nutr Res. (2014) 34:577–84. doi: 10.1016/j.nutres.2014.07.002, PMID: 25150116

[37] van der Pol A van Gilst WH Voors AA van der Meer P. Treating oxidative stress in heart failure: past, present and future. Eur J Heart Fail. (2019) 21:425–35. doi: 10.1002/ejhf.1320, PMID: 30338885 PMC6607515

[38] Fukumoto LR Mazza G. Assessing antioxidant and prooxidant activities of phenolic compounds. J Agric Food Chem. (2000) 48:3597–604. doi: 10.1021/jf000220w, PMID: 10956156

[39] Mortensen RM. Immune cell modulation of cardiac remodeling. Circulation. (2012) 125:1597–600. doi: 10.1161/circulationaha.112.097832, PMID: 22388322

[40] Hou DX Kai K Li JJ Lin S Terahara N Wakamatsu M. Anthocyanidins inhibit activator protein 1 activity and cell transformation: structure-activity relationship and molecular mechanisms. Carcinogenesis. (2004) 25:29–36. doi: 10.1093/carcin/bgg184, PMID: 14514663

[41] Min SW Ryu SN Kim DH. Anti-inflammatory effects of black rice, cyanidin-3-O-beta-D-glycoside, and its metabolites, cyanidin and protocatechuic acid. Int Immunopharmacol. (2010) 10:959–66. doi: 10.1016/j.intimp.2010.05.009, PMID: 20669401

[42] Hou DX Yanagita T Uto T Masuzaki S Fujii M. Anthocyanidins inhibit cyclooxygenase-2 expression in LPS-evoked macrophages: structure-activity relationship and molecular mechanisms involved. Biochem Pharmacol. (2005) 70:417–25. doi: 10.1016/j.bcp.2005.05.003, PMID: 15963474

[43] Chen L Teng H Fang T Xiao J. Agrimonolide from Agrimonia pilosa suppresses inflammatory responses through down-regulation of COX-2/iNOS and inactivation of NF-κB in lipopolysaccharide-stimulated macrophages. Phytomedicine. (2016) 23:846–55. doi: 10.1016/j.phymed.2016.03.016, PMID: 27288920

[44] Aboonabi A Aboonabi A. Anthocyanins reduce inflammation and improve glucose and lipid metabolism associated with inhibiting nuclear factor-kappaB activation and increasing PPAR-γ gene expression in metabolic syndrome subjects. Free Radic Biol Med. (2020) 150:30–9. doi: 10.1016/j.freeradbiomed.2020.02.004, PMID: 32061902

[45] Seeram NP. Berry fruits: compositional elements, biochemical activities, and the impact of their intake on human health, performance, and disease. J Agric Food Chem. (2008) 56:627–9. doi: 10.1021/jf071988k, PMID: 18211023

[46] Heneghan C Kiely M Lyons J Lucey A. The effect of Berry-based food interventions on markers of cardiovascular and metabolic health: a systematic review of randomized controlled trials. Mol Nutr Food Res. (2018) 62. doi: 10.1002/mnfr.201700645, PMID: 29105295

[47] Kalt W Cassidy A Howard LR Krikorian R Stull AJ Tremblay F. Recent research on the health benefits of blueberries and their Anthocyanins. Adv Nutr. (2020) 11:224–36. doi: 10.1093/advances/nmz065, PMID: 31329250 PMC7442370

[48] Blumberg JB Camesano TA Cassidy A Kris-Etherton P Howell A Manach C. Cranberries and their bioactive constituents in human health. Adv Nutr. (2013) 4:618–32. doi: 10.3945/an.113.004473, PMID: 24228191 PMC3823508

