# Supplementary material for: Association between dietary intake of anthocyanidins and heart failure among American adults: NHANES (2007–2010 and 2017–2018)
Source: Front Nutr. 2023 Apr 5;10:1107637. doi: 10.3389/fnut.2023.1107637 (PMC10113463; doi:10.3389/fnut.2023.1107637)
Supplement: Supplementary file 1 [file Table_1.docx]

supplement table 2 univariate logistic regression analyses

| character | Estimate | Std. Error | t value | Pr(>\|t\|) | OR | 95% CI |
| --- | --- | --- | --- | --- | --- | --- |
| Total_Anthocyanidins_mgQ |  |  |  |  |  |  |
| Q1 | ref | ref | ref | ref | ref | ref |
| Q2 | -0.08 | 0.18 | -0.48 | 0.63 | 0.92 | 0.92(0.64,1.31) |
| Q3 | -0.04 | 0.16 | -0.28 | 0.78 | 0.96 | 0.96(0.70,1.31) |
| Q4 | -0.49 | 0.14 | -3.53 | <0.001 | 0.61 | 0.61(0.46,0.81) |
| Total_Anthocyanidins, mg | -0.01 | 0 | -2.67 | 0.01 | 0.99 | 0.99(0.99,1.00) |
| Gender |  |  |  |  |  |  |
| Male | ref | ref | ref | ref | ref | ref |
| Female | -0.33 | 0.1 | -3.26 | 0.002 | 0.72 | 0.72(0.58,0.88) |
| Race |  |  |  |  |  |  |
| White | ref | ref | ref | ref | ref | ref |
| Black | 0.28 | 0.14 | 1.97 | 0.05 | 1.32 | 1.32(0.99,1.75) |
| Mexican | -0.96 | 0.18 | -5.46 | <0.0001 | 0.38 | 0.38(0.27,0.55) |
| Other | -0.28 | 0.18 | -1.56 | 0.13 | 0.75 | 0.75(0.52,1.09) |
| Smoke |  |  |  |  |  |  |
| No | ref | ref | ref | ref | ref | ref |
| Yes | 0.7 | 0.13 | 5.38 | <0.0001 | 2.02 | 2.02(1.55,2.63) |
| Age | 0.07 | 0 | 23.08 | <0.0001 | 1.08 | 1.08(1.07,1.08) |
| BMI, kg/m2 | 0.06 | 0.01 | 10.28 | <0.0001 | 1.06 | 1.06(1.05,1.07) |
| Waist, cm | 0.04 | 0 | 11.52 | <0.0001 | 1.04 | 1.04(1.03,1.04) |
| LDL cholesterol, mg/dl | -0.01 | 0 | -2.32 | 0.02 | 0.99 | 0.99(0.98,1.00) |
| HDL cholesterol, mg/dl | -0.02 | 0.01 | -4.48 | <0.0001 | 0.98 | 0.98(0.97,0.99) |
| Total_cholesterol, mg/dl | -0.01 | 0 | -4.74 | <0.0001 | 0.99 | 0.99(0.98,0.99) |
| Triglycerides, mg/dl | 0 | 0 | 4.53 | <0.0001 | 1 | 1.00(1.00,1.00) |
| Fast glucose, mmol/L | 0.18 | 0.02 | 7.67 | <0.0001 | 1.19 | 1.19(1.14,1.25) |
| HbA1c, g/dl | 0.44 | 0.03 | 16.14 | <0.0001 | 1.55 | 1.55(1.47,1.64) |
| eGFR, mL/min/1.73 m2 | -0.05 | 0 | -17.83 | <0.0001 | 0.95 | 0.95(0.95,0.96) |
| Creatinine, mg/dl | 0.77 | 0.17 | 4.65 | <0.0001 | 2.17 | 2.17(1.55,3.03) |
| DM |  |  |  |  |  |  |
| DM | ref | ref | ref | ref | ref | ref |
| IFG | -1.76 | 0.42 | -4.16 | <0.001 | 0.17 | 0.17(0.07,0.40) |
| IGT | -1.02 | 0.27 | -3.75 | <0.001 | 0.36 | 0.36(0.21,0.63) |
| No | -2.02 | 0.11 | -18.04 | <0.0001 | 0.13 | 0.13(0.11,0.17) |
| Hypertension |  |  |  |  |  |  |
| No | ref | ref | ref | ref | ref | ref |
| Yes | 2.01 | 0.16 | 12.2 | <0.0001 | 7.46 | 7.46(5.36,10.40) |
| Hyperlipidemia |  |  |  |  |  |  |
| No | ref | ref | ref | ref | ref | ref |
| Yes | 0.96 | 0.17 | 5.71 | <0.0001 | 2.62 | 2.62(1.86,3.67) |
| ACE inhibitors |  |  |  |  |  |  |
| No | ref | ref | ref | ref | ref | ref |
| Yes | 0.12 | 0.31 | 0.39 | 0.70 | 1.13 | 1.13(0.60,2.12) |
| Beta blocker |  |  |  |  |  |  |
| No | ref | ref | ref | ref | ref | ref |
| Yes | 2.84 | 0.11 | 24.91 | <0.0001 | 17.06 | 17.06(13.56,21.45) |
| Diuretics |  |  |  |  |  |  |
| No | ref | ref | ref | ref | ref | ref |
| Yes | 2.38 | 0.12 | 20.72 | <0.0001 | 10.83 | 10.83(8.59,13.66) |
| Statin |  |  |  |  |  |  |
| No | ref | ref | ref | ref | ref | ref |
| Yes | 1.91 | 0.12 | 15.58 | <0.0001 | 6.74 | 6.74(5.27,8.62) |
| Coronary.Heart.Disease |  |  |  |  |  |  |
| No | ref | ref | ref | ref | ref | ref |
| Yes | 3.22 | 0.13 | 25.05 | <0.0001 | 24.97 | 24.97(19.28,32.33) |
| Smoke |  |  |  |  |  |  |
| Former | ref | ref | ref | ref | ref | ref |
| Never | -0.97 | 0.14 | -6.85 | <0.0001 | 0.38 | 0.38(0.29,0.50) |
| Now | -0.73 | 0.14 | -5.07 | <0.0001 | 0.48 | 0.48(0.36,0.64) |
