# Supplementary material for: Association between dietary intake of anthocyanidins and heart failure among American adults: NHANES (2007–2010 and 2017–2018)
Source: Front Nutr. 2023 Apr 5;10:1107637. doi: 10.3389/fnut.2023.1107637 (PMC10113463; doi:10.3389/fnut.2023.1107637)
Supplement: Supplementary file 2 [file Table_2.DOCX]

Supplement Table 2: The intake of anthocyanidins between HF and non-HF for each quartile (Q2-4).

| Group | Total | Non-heart Failure | Heart failure | *P* value |
| --- | --- | --- | --- | --- |
| Q2 | 0.24(0.22,0.25) | 0.23(0.22,0.25) | 0.26(0.19,0.33) | 0.49 |
| Q3 | 2.82(2.75,2.88) | 2.82(2.75,2.89) | 2.70(2.46,2.94) | 0.36 |
| Q4 | 47.59(44.47,50.71) | 47.75(44.62,50.88) | 37.72(29.19,46.25) | 0.02 |
