# Supplementary figures and images for: Association between dietary intake of anthocyanidins and heart failure among American adults: NHANES (2007–2010 and 2017–2018)
Source: Front Nutr. 2023 Apr 5;10:1107637. doi: 10.3389/fnut.2023.1107637 (PMC10113463; doi:10.3389/fnut.2023.1107637)

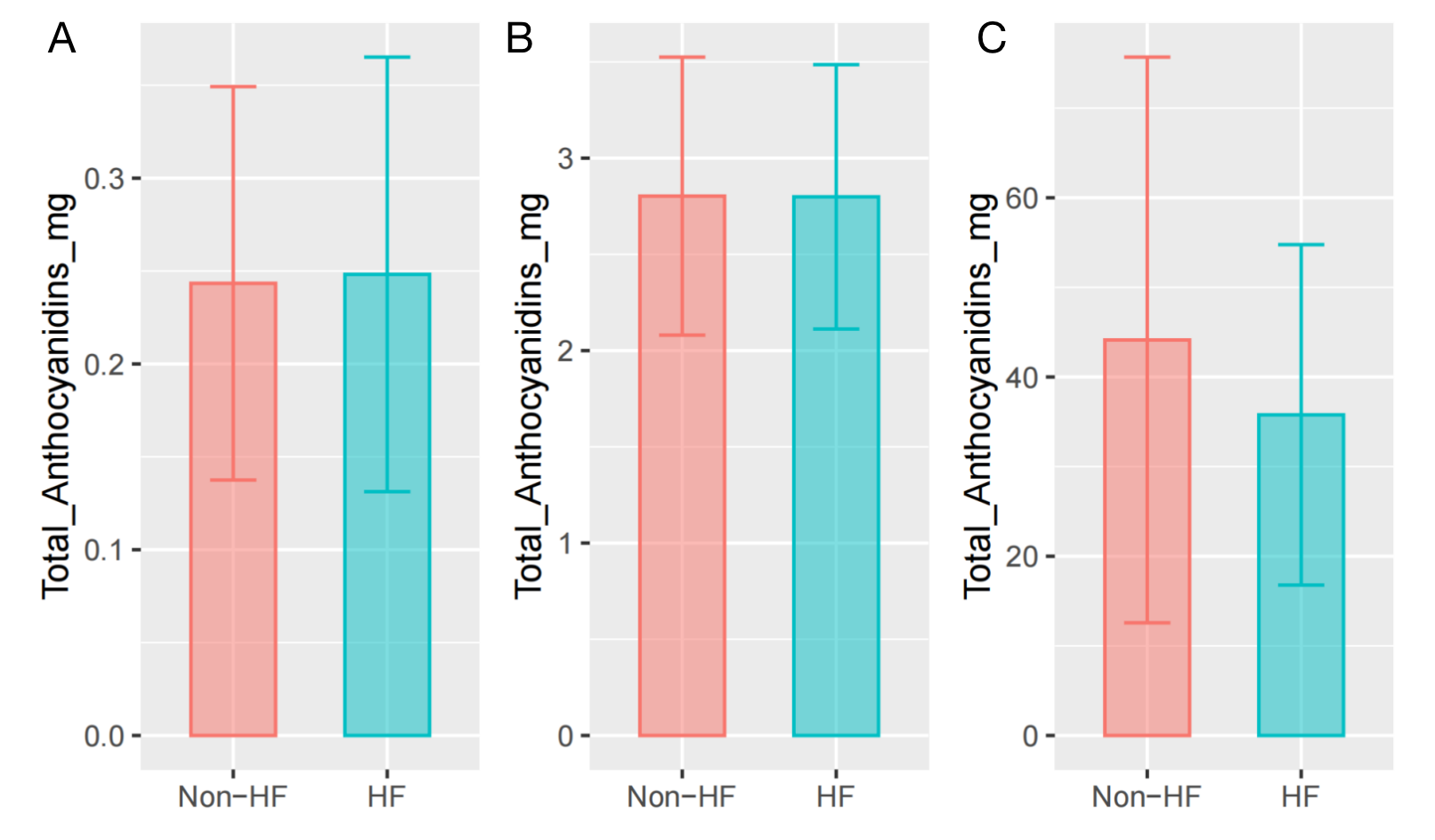

Supplement: Supplementary file 3 [file Image_1.PNG]
